# *Histoplasma* Urinary Antigen for the Diagnosis Histoplasmosis in Non‐HIV Individuals

**DOI:** 10.1111/myc.70183

**Published:** 2026-05-05

**Authors:** João Nobrega de Almeida, Tamara Harb Roca, Denise dos Anjos Laurentis de Souza Campos, Renée Zon Filippi, Cristóvão Luis P. Mangueira, Marines Dalla Valle Martino

**Affiliations:** ^1^ Einstein Hospital Israelita São Paulo Brazil; ^2^ Federal University of São Paulo São Paulo Brazil

**Keywords:** antigen, diagnosis, histoplasmosis, HIV‐negative patients

## Abstract

**Background:**

Histoplasmosis is an endemic mycosis in the Americas. While urinary *Histoplasma* antigen enzyme immunoassay testing (HAET) has been validated mainly in HIV‐infected patients with disseminated disease, its performance in HIV‐negative populations with heterogeneous clinical presentations is unclear.

**Objectives:**

To describe the performance of urinary HAET for the diagnosis of histoplasmosis in a cohort of Brazilian HIV‐negative patients.

**Material and Methods:**

We conducted a retrospective study at Einstein Hospital Israelita (Brazil) including HIV‐negative patients whose urine samples were analysed by *Histoplasma* antigen enzyme immunoassay test (HAET) from January 2022 to October 2025. Factors associated with positive urinary HAET were analysed using univariable and multivariable models. Among 346 patients, histoplasmosis was diagnosed in 43 (12%); 16 (37%) were immunocompromised and 14 (33%) had disseminated disease. Urinary HAET was positive in eight patients (19%), with antigen levels from 0.3 to > 15 ng/mL. Sensitivity and specificity were 19% and 100%, respectively; positive and negative predictive values were 100% and 90%. Sensitivity increased to 45% in immunocompromised patients with multiorgan involvement, and immunocompromised status independently predicted HAET positivity (*p* = 0.008). In HIV‐negative patients, HAET demonstrates excellent specificity but low sensitivity, performing best in immunocompromised individuals with disseminated histoplasmosis.

## 1 Introduction

Histoplasmosis is an endemic mycosis in the Americas, with clinical presentations of acute forms ranging from self‐limited pulmonary disease to severe disseminated forms, especially in immunocompromised individuals [1]. Traditional and non‐invasive laboratory diagnosis is based on culture and serological tests, which may fail in early disease stages or in immunocompromised patients [2]. Fungal antigen detection assays in urine targeting cell wall polysaccharides have revolutionized the diagnosis of histoplasmosis [3]. However, most of the established performance evidence has been generated in HIV‐infected populations [3], leaving gaps in non‐HIV subgroups, such as transplant recipients, patients using corticosteroids or immunobiologics, those with malignancies, and immunocompetent hosts.

In the non‐HIV context, the clinical presentation of histoplasmosis is heterogeneous [2]. In these scenarios, the sensitivity of the urine antigen test may be lower than that observed in progressive disseminated disease in people with HIV with a high fungal burden [3]. In addition, potential cross‐reactions (e.g., with Blastomycosis) may reduce specificity in regions endemic for other mycoses [3]. In Brazil, where histoplasmosis is endemic, there is a scarcity of data regarding the diagnostic performance of urinary antigen in non‐HIV individuals [4]. Therefore, a focused study evaluating the performance of the urinary *Histoplasma* Antigen enzyme immunoassay test (HAET) in this population will help to fill this knowledge gap, create diagnostic algorithms, and guide rational use and interpretation of this test in routine clinical practice.

## 2 Material and Methods

This retrospective single‐centre study was conducted at the Einstein Hospital Israelita (EHI), from São Paulo, Brazil. The EHI is a private referral centre and receives patients from various cities in Brazil and other South American countries. HIV‐negative patients with urine samples submitted for HAET (Clarus Histoplasma Galactomannan EIA, IMMY, Oklahoma, USA), from January 2022 to October 2025 were enrolled. HAET results were provided by Quest Diagnostics (Secaucus, New Jersey, USA) and expressed in ng/mL, with values ranging from < 0.2 ng/mL (negative) to 0.2 to > 15 ng/mL (positives). Detailed medical records were reviewed at the EHI with the approval of the institutional review board (n. CAAE 95660326.0.0000.0071.).

Clinical data were collected using a standardized Excel file and included information on the clinical syndrome, clinical and laboratory criteria supporting the diagnosis, and immune status. Patients with haematological malignancies, or that underwent solid organ transplantation, or receiving high dose corticosteroids (≥ 20 mg of prednisone or equivalent, for at least 2 weeks [5]) or TNF antagonists were considered immunocompromised. Patients with the involvement of more than one organ (e.g., lung and liver) were classified as having disseminated histoplasmosis. After data collection, all participants were de‐identified, and the anonymized data were used for analysis. Cases with missing clinical data were excluded from the study.

Patients were classified as either having proven or probable histoplasmosis according to a previous definition [3]. Histoplasma antibody tests demonstrating H or M precipitin bands by immunodiffusion (ID) and/or titers of complement fixation (CF) antibodies of ≥ 1:8 were considered positive for anti‐*Histoplasma* antibody [3]. Histoplasma antibody tests were conducted at Quest Diagnostics (Secaucus, New Jersey, USA) using a household assay validated in accordance with CLIA regulations.

Pulmonary histoplasmosis was diagnosed in patients who had respiratory symptoms plus chest radiographs and/or computed tomography scans showing pulmonary infiltrates and/or mediastinal lymphadenopathy. Pulmonary cases were then categorized as acute, subacute, or chronic, as previously described [3]. Asymptomatic patients with solitary pulmonary nodules found accidentally during investigation of other possible pulmonary diseases (e.g., lung metastases investigation) were classified as having a “solitary histoplasmoma.”

To describe the potential variables associated with a positive urinary HAET, data comparisons between the positive vs. non‐positive patients were carried out with SPSS software v.22 (IBM, Armonk, NY, USA). Categorical variables were expressed as percentage and continuous variables as median ± standard deviation (SD). Differences between the groups were evaluated with Chi‐squared test, Fisher's exact test or the Mann–Whitney *U* test. Variables associated with *p* values < 0.1 on univariable basis were introduced into the multivariable model. Two‐tailed *p* values < 0.05 were considered statistically significant.

## 3 Results

During the study period, 374 urine samples were sent for HAET analysis. Four samples from three patients were excluded from the analysis due to the lack of clinical data. Thus, 370 samples from 346 non‐HIV patients were analyzed.

**FIGURE 1:**
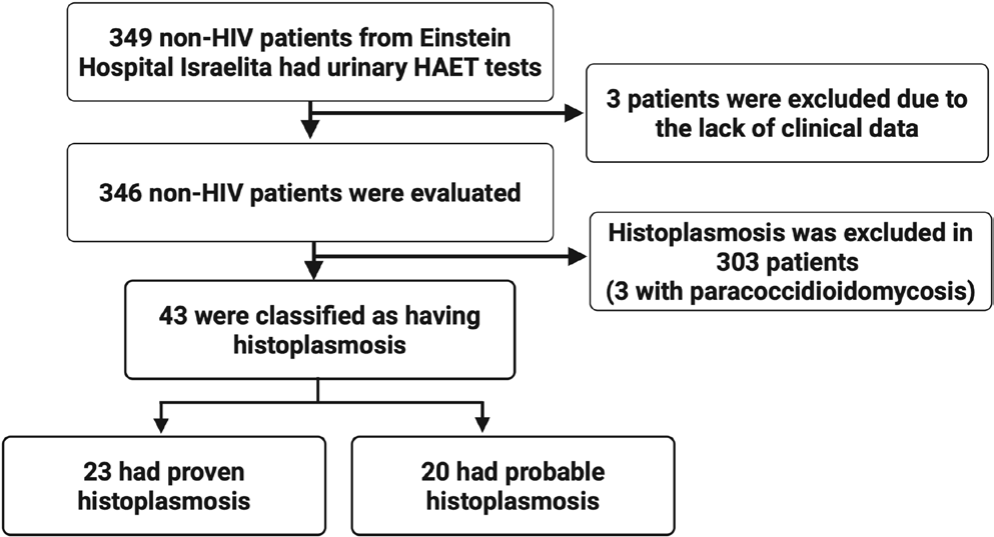
Flowchart showing patient selection and classification.

A total of 43 (12%) patients were classified as having either proven (*n* = 23, 53%) or probable histoplasmosis (*n* = 20, 47%). A flowchart illustrating patient selection and classification is provided in Figure 1. Among the 43 patients with histoplasmosis, 16 (37%) were considered immunocompromised, from which six (38%) had a solid organ transplant (kidney *n* = 4, heart *n* = 1, liver *n* = 1). Disseminated disease accounted for 14 (33%) cases, while 29 had only pulmonary involvement (77%). Among the pulmonary cases, acute or subacute cases accounted for 24 cases (83%), while solitary histoplasmoma was detected in five cases (17%).

Eight urine samples from different patients were positive (2%). The positive HAET results varied from 0.3 to > 15 ng/mL. The characteristics of the patients with positive HAET tests are summarized in Table 1. The characteristics and comparisons of the 43 histoplasmosis patients with positive or negative urinary HAET tests are summarized in Table 2. The urinary HAET test performance for the diagnosis of histoplasmosis in this non‐HIV cohort varied according to the clinical presentation and immune status of the patients (Table 2).

**TABLE 1:** Patients with positive *Histoplasma* urinary antigen.

Case	Age/Sex	Baseline disease	Immunosuppression	Clinical features	Symptom duration (days)	Disease classification	Clinical presentation	Antigen value (ng/mL)	Serology	Culture
1	56/F	Psoriatic arthritis	Adalimumab, methotrexate	Fever, arthralgia, respiratory symptoms, skin lesions	21	Proven	Disseminated	0.3	Negative	Positive
2	43/M	Kidney transplant	Tacrolimus, Methotrexate	Fever, abdominal pain	14	Proven	Disseminated	> 15	Negative	Positive
3	35/M	Kidney transplant	Tacrolimus, Methotrexate	Fever, respiratory symptoms, abdominal pain	29	Proven	Disseminated	> 15	Positive	Positive
4	44/M	Liver transplant	Tacrolimus	Arthralgia, Abdominal pain, oral ulcer.	30	Proven	Disseminated	0.7	Positive	Negative
5	46/M	Psoriasis	Methotrexate	Fever, respiratory symptoms	3	Proven	Acute pulmonary	0.9	Negative	Negative
6	36/M	Kidney transplant	Tacrolimus, High dose steroids	Fever, respiratory symptoms, abdominal pain, oral ulcer	13	Proven	Disseminated	14.6	Negative	Negative
7	66/M	None (False diagnosis of rheumatoid arthritis)	Golimumab, High dose steroids	Fever, arthralgia, respiratory symptoms, skin lesions	20	Probable	Disseminated	0.8	Positive	Negative
8	46/M	None	None	Respiratory symptoms	5	Probable	Acute pulmonary	3.5	Positive	Not done

**TABLE 2:** Main characteristics of 43 histoplasmosis patients according to antigen detection.

Characteristic	Positive cases (*n* = 8)	Negative cases (*n* = 35)	*p*
Demographics			
Age (mean, ±SD)	45 ± 9.6	51 ± 14.9	NS
Sex (female, %)	1 (13%)	15 (43%)	NS
Immune status (*n*, %)			
Immunocompromised	6 (75%)	2 (6%)	< 0.001
Non‐immunocompromised	2 (25%)	33 (94%)	
Clinical form (*n*, %)			
Disseminated	6 (75%)	8 (21%)	0.01
Acute or subacute pulmonary	2 (25%)	22 (65%)	
Solitary histoplasmoma	0 (%)	5 (14%)	
Diagnosis classification (*n*, %)			
Proven	5 (63%)	18 (51%)	NS
Probable	3 (37%)	17 (49%)	

Abbreviation: NS, not statistically significant.

The overall sensitivity of the HAET test was 19%, while the specificity was 100%. Of note, all three patients with paracoccidioidomycosis had negative HAET tests. The positive and negative predictive values were 100% and 90%, respectively. A sensitivity of 45% was seen in immunosuppressed patients with multiorgan involvement.

In multivariable analysis, immunocompromised patients had higher odds of testing positive for the urinary antigen (*p* = 0.008, OR 20.222, 95% CI 2.178–187.723).

## 4 Discussion

Our findings corroborate that urine *Histoplasma* antigen testing in HIV‐negative patients without disseminated disease has excellent specificity but limited overall sensitivity, aligning with and extending observations from prior cohorts [1, 3]. In our study, overall sensitivity was 19%, rising to 45% among immunocompromised patients with multiorgan involvement. By contrast, cohorts in HIV‐infected individuals with disseminated histoplasmosis have reported sensitivities of 80%–95% and specificities typically above 90%, reflecting possibly the higher fungal burden and more uniform disseminated presentation in that population [3].

In non‐HIV settings, published data are more heterogeneous and generally indicate lower sensitivity, particularly in localized forms [6]. Studies in solid organ transplant recipients, rheumatologic patients receiving biologics, and those with hematologic malignancies have shown sensitivities ranging from 30% to 93%, often highest in disseminated disease and lowest in isolated pulmonary involvement or solitary nodules [7, 8]. The 45% sensitivity we observed in immunocompromised patients with multiorgan disease is consistent with these reports, suggesting that fungal burden and tissue dissemination remain key determinants of antigen detectability, even outside the HIV context.

Our cohort was notable for a predominance of pulmonary disease (77%), including solitary histoplasmomas, in which antigenuria is expected to be low. This likely contributed to the reduced overall sensitivity. In contrast, cohorts enriched for severe disseminated disease tend to report higher test performance [3]. Importantly, we did not observe cross‐reactivity with paracoccidioidomycosis in the small number of tested cases, whereas prior series from the USA have highlighted clinically relevant cross‐reactions with blastomycosis [3].

Our results emphasize the need for context‐specific interpretation, integrating antigen testing with imaging, serology, microbiological, and histopathological evidence [5]. Finally, the Brazilian setting and inclusion of diverse immunosuppressive conditions add regionally relevant data to the literature and underscore the importance of local validation studies to guide diagnostic algorithms in non‐HIV populations.

This study is limited by its retrospective single‐centre design and a relatively small number of histoplasmosis cases, restricting the precision and generalizability of its performance estimates.

## Author Contributions

**João Nobrega de Almeida Jr:** conceptualization, investigation, writing – original draft, methodology, visualization, writing – review and editing, data curation, supervision. **Tamara Harb Roca:** investigation, writing – original draft, methodology, validation, writing – review and editing, data curation. **Renée Zon Filippi:** investigation, methodology, visualization, data curation, resources. **Cristóvão Luis P. Mangueira:** investigation, validation, visualization, writing – review and editing, project administration, supervision. **Denise dos Anjos Laurentis de Souza Campos:** investigation, methodology, data curation, resources. **Marines Dalla Valle Martino:** investigation, validation, visualization, writing – review and editing, project administration, supervision.

## Conflicts of Interest

The authors declare no conflicts of interest.

## Data Availability

The data that support the findings of this study are available from the corresponding author upon reasonable request.
